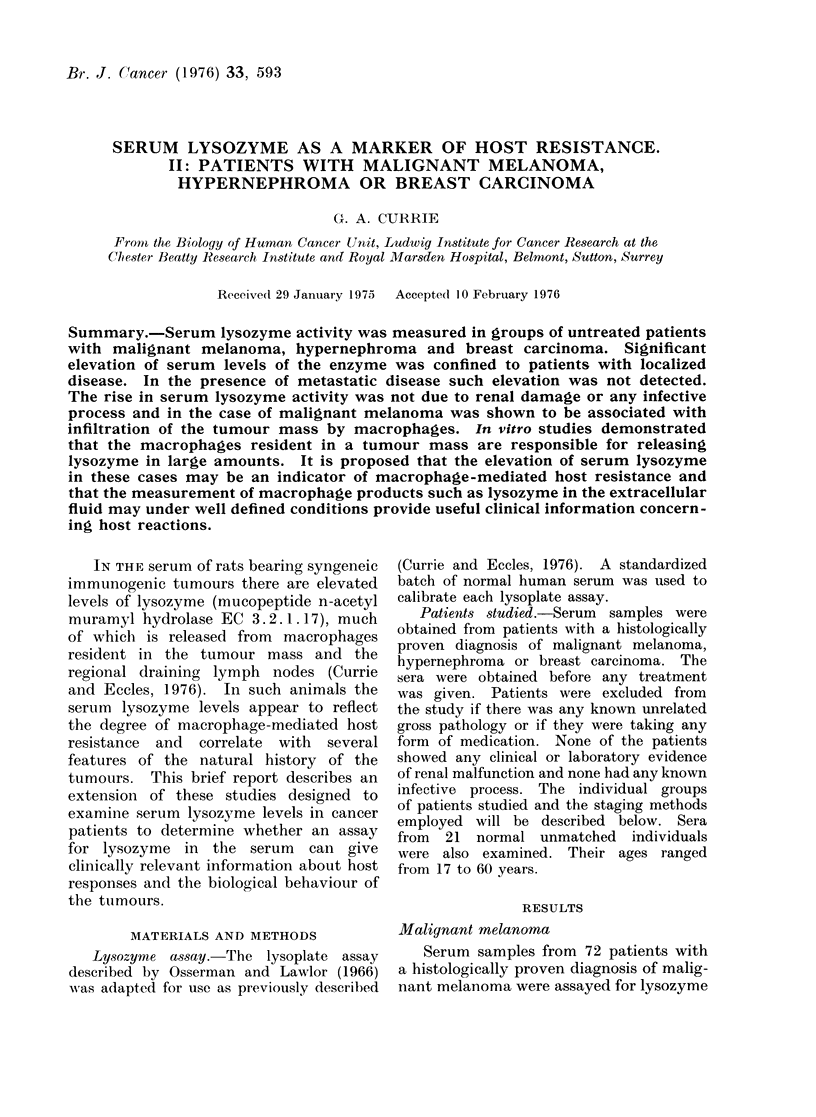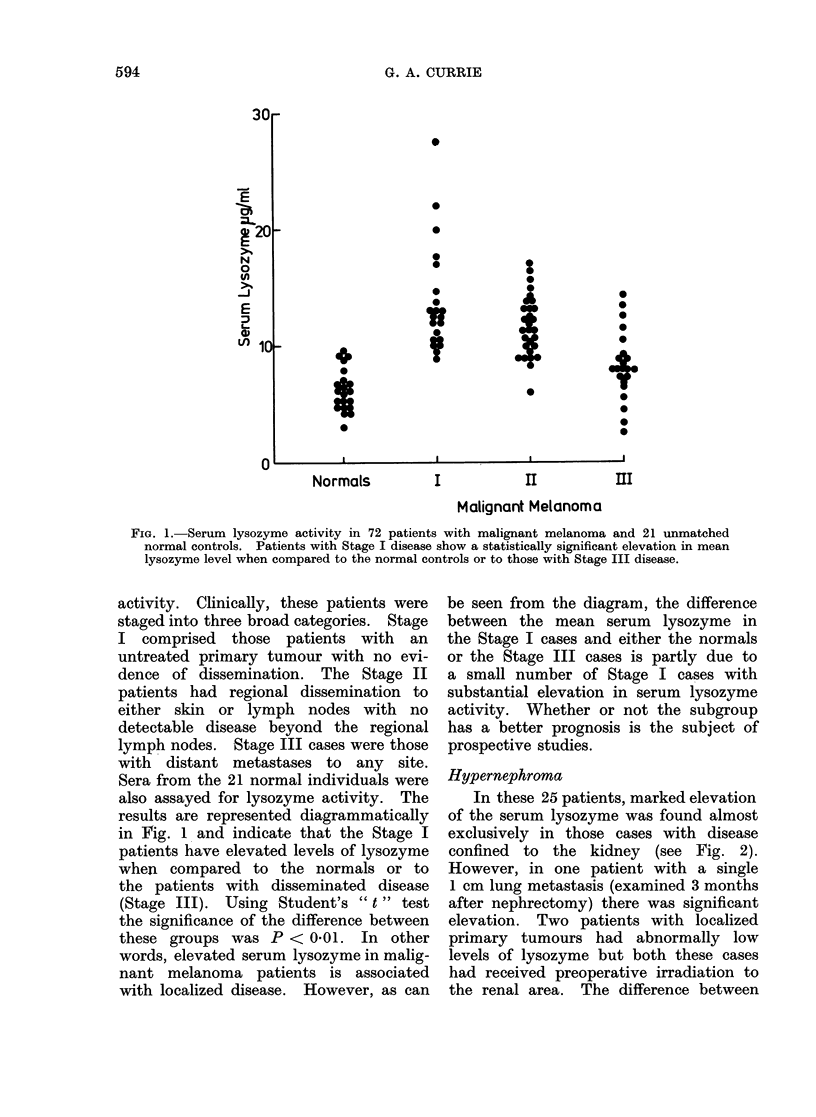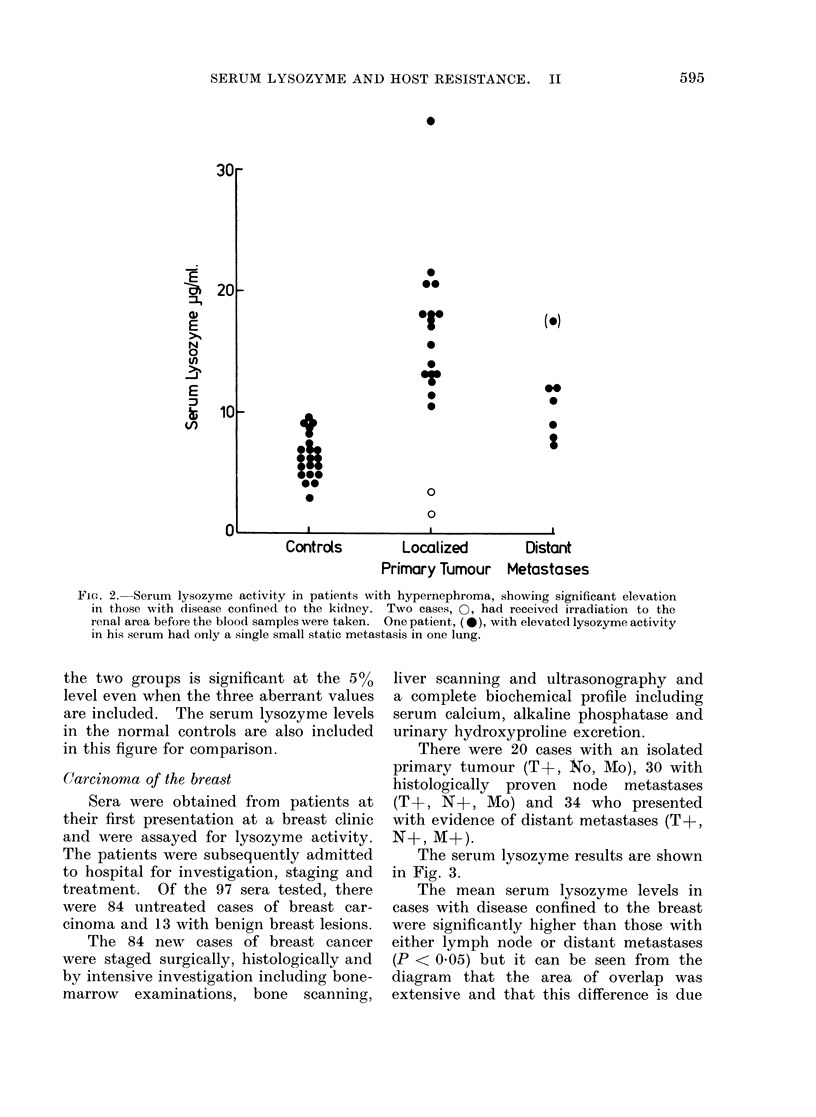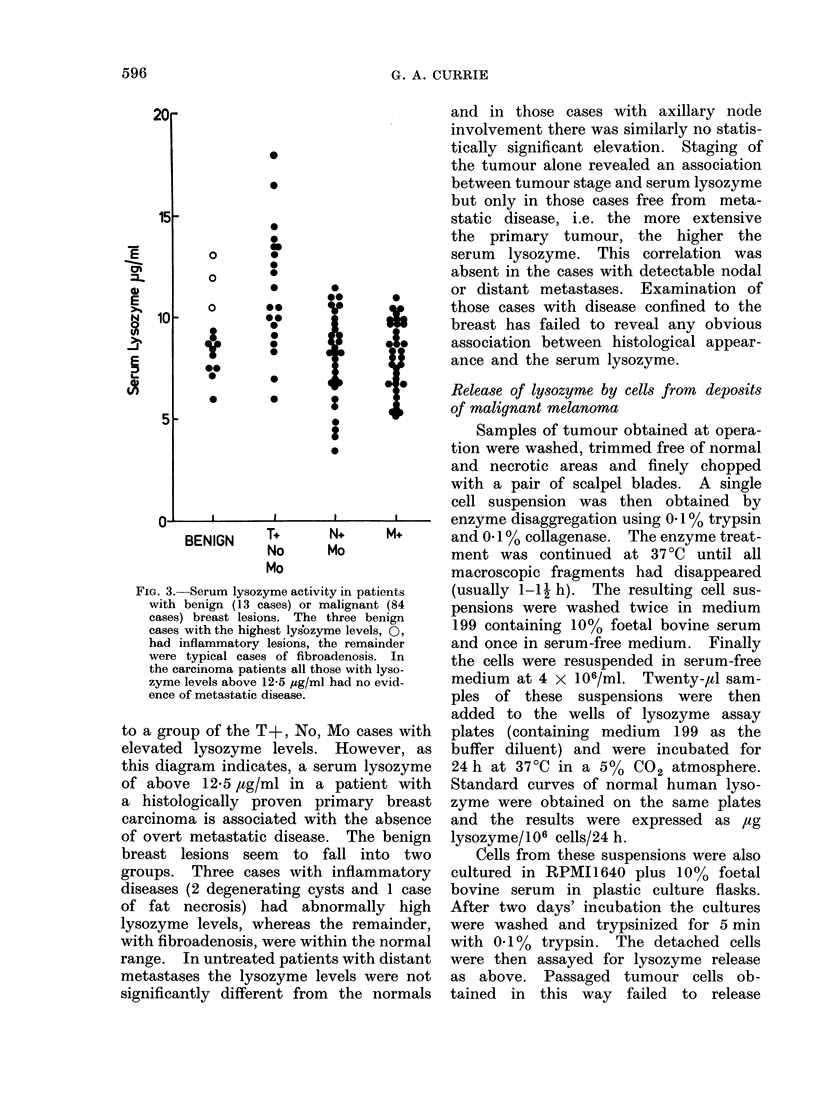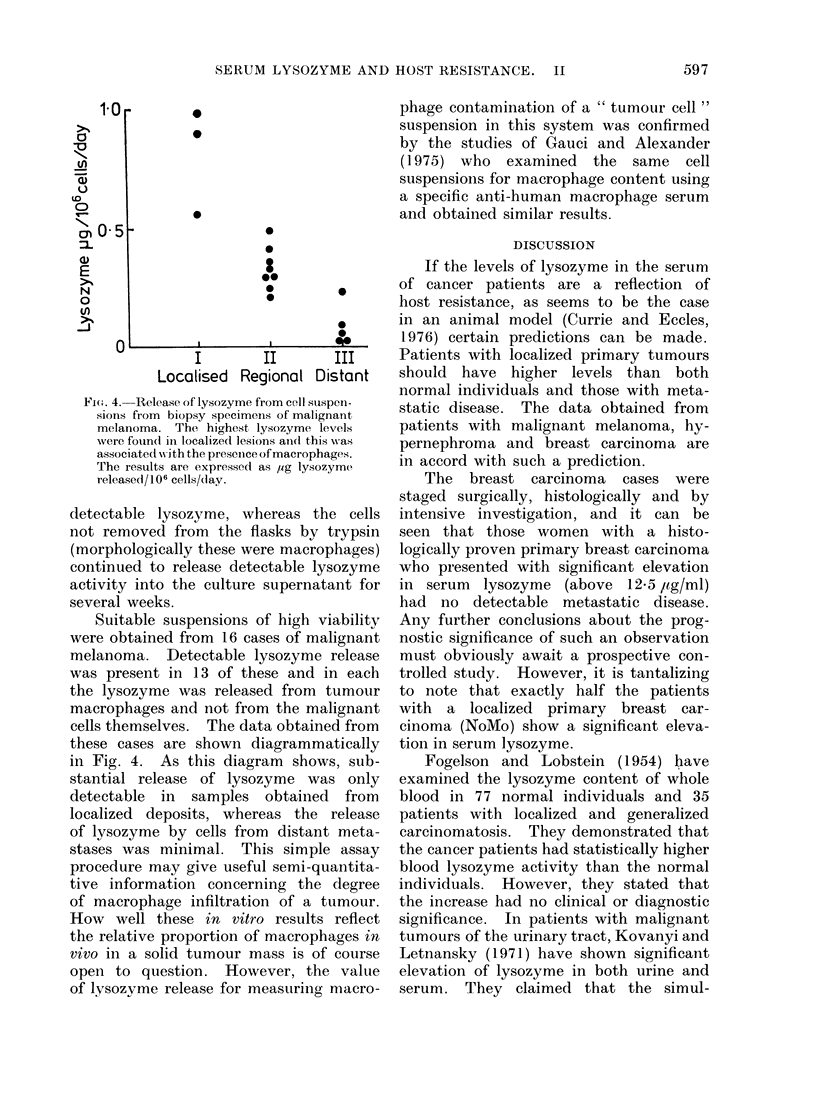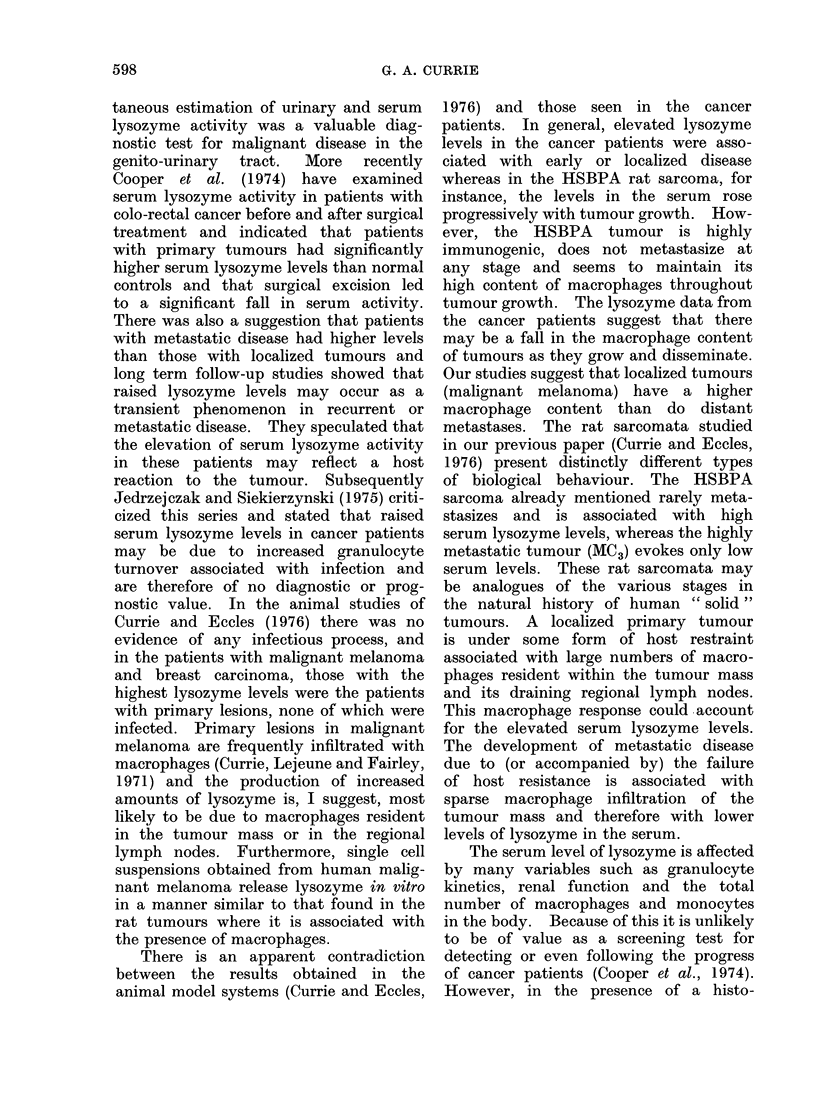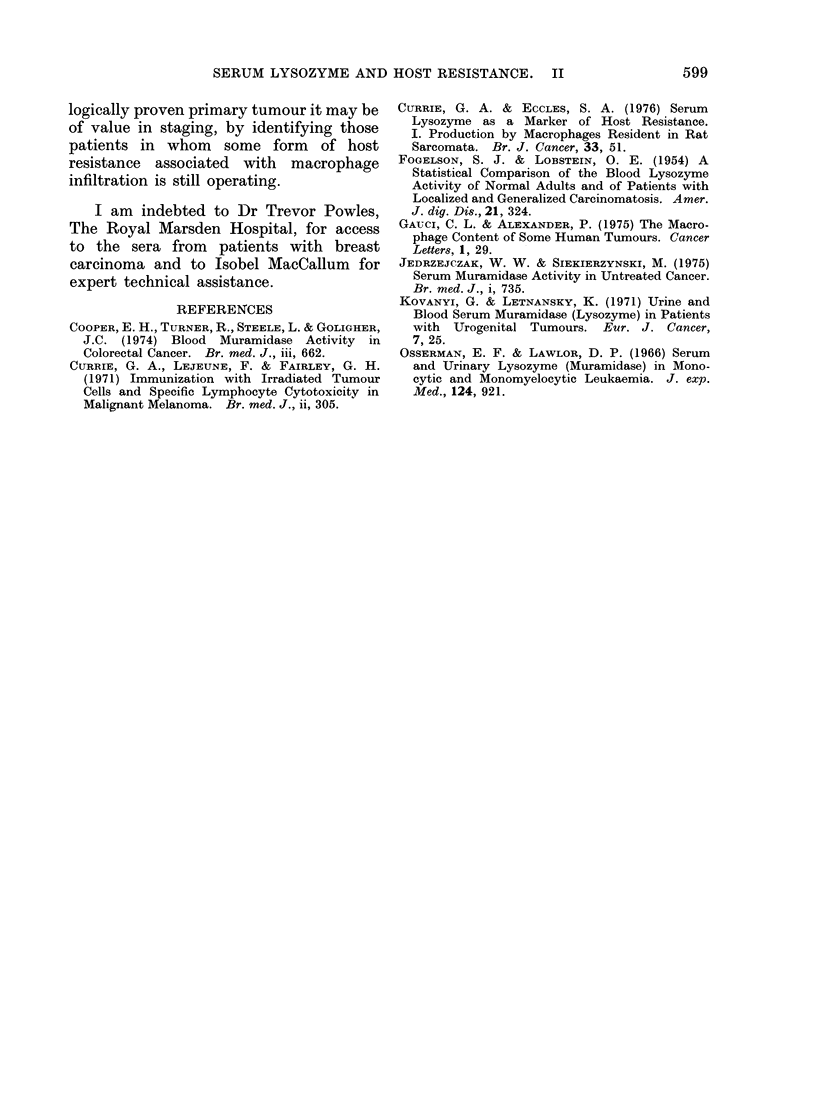# Serum lysozyme as a marker of host resistance. II. Patients with malignant melanoma, hypernephroma or breast carcinoma.

**DOI:** 10.1038/bjc.1976.96

**Published:** 1976-06

**Authors:** G. A. Currie

## Abstract

Serum lysozyme activity was measured in groups of untreated patients with malignant melanoma, hyperneophroma and breast carcinoma. Significant elevation of serum levels of the enzyme was confined to patients with localized disease. In the presence of metastatic disease such elevation was not detected. The rise in serum lysozyme activity was not due to renal damage or any infective process and in the case of malignant melanoma was shown to be associated with infiltration of the tumour mass by macrophages. In vitro studies demonstrated that the macrophages resident in a tumour mass are responsible for relasing lysozyme in large amounts. It is proposed that the elevation of serum lysozyme in these cases may be an indicator of macrophage-mediated host resistance and that the measurement of macrophage products such as lysozyme in the extracellular fluid may under well defined conditions provide useful clinical information concerning host reactions.


Br. J. Cancer (1976) 33, 593

### SERUM LYSOZYME AS A MARKER OF HOST RESISTANCE. II: PATIENTS WITH MALIGNANT MELANOMA, HYPERNEPHROMA OR BREAST CARCINOMA

G. A. CURRIE

*From the Biology of Human Cancer Unit, Ludwig Institute for Cancer Research at the Chester Beatty Research Institute and Royal Marsden Hospital, Belmont, Sutton, Surrey*

Received 29 January 1975 Accepted 10 February 1976

**Summary.—Serum lysozyme activity was measured in groups of untreated patients with malignant melanoma, hypernephroma and breast carcinoma. Significant elevation of serum levels of the enzyme was confined to patients with localized disease. In the presence of metastatic disease such elevation was not detected. The rise in serum lysozyme activity was not due to renal damage or any infective process and in the case of malignant melanoma was shown to be associated with infiltration of the tumour mass by macrophages. *In vitro* studies demonstrated that the macrophages resident in a tumour mass are responsible for releasing lysozyme in large amounts. It is proposed that the elevation of serum lysozyme in these cases may be an indicator of macrophage-mediated host resistance and that the measurement of macrophage products such as lysozyme in the extracellular fluid may under well defined conditions provide useful clinical information concerning host reactions.**

IN THE serum of rats bearing syngeneic immunogenic tumours there are elevated levels of lysozyme (mucopeptide n-acetyl muramyl hydrolase EC 3.2.1.17), much of which is released from macrophages resident in the tumour mass and the regional draining lymph nodes (Currie and Eccles, 1976). In such animals the serum lysozyme levels appear to reflect the degree of macrophage-mediated host resistance and correlate with several features of the natural history of the tumours. This brief report describes an extension of these studies designed to examine serum lysozyme levels in cancer patients to determine whether an assay for lysozyme in the serum can give clinically relevant information about host responses and the biological behaviour of the tumours.

### MATERIALS AND METHODS

*Lysozyme assay.*—The lysoplate assay described by Osserman and Lawlor (1966) was adapted for use as previously described (Currie and Eccles, 1976). A standardized batch of normal human serum was used to calibrate each lysoplate assay.

*Patients studied.*—Serum samples were obtained from patients with a histologically proven diagnosis of malignant melanoma, hypernephroma or breast carcinoma. The sera were obtained before any treatment was given. Patients were excluded from the study if there was any known unrelated gross pathology or if they were taking any form of medication. None of the patients showed any clinical or laboratory evidence of renal malfunction and none had any known infective process. The individual groups of patients studied and the staging methods employed will be described below. Sera from 21 normal unmatched individuals were also examined. Their ages ranged from 17 to 60 years.

### RESULTS

### *Malignant melanoma*

Serum samples from 72 patients with a histologically proven diagnosis of malignant melanoma were assayed for lysozyme

FIG. 1.—Serum lysozyme activity in 72 patients with malignant melanoma and 21 unmatched normal controls. Patients with Stage I disease show a statistically significant elevation in mean lysozyme level when compared to the normal controls or to those with Stage III disease.

activity. Clinically, these patients were staged into three broad categories. Stage I comprised those patients with an untreated primary tumour with no evidence of dissemination. The Stage II patients had regional dissemination to either skin or lymph nodes with no detectable disease beyond the regional lymph nodes. Stage III cases were those with distant metastases to any site. Sera from the 21 normal individuals were also assayed for lysozyme activity. The results are represented diagrammatically in Fig. 1 and indicate that the Stage I patients have elevated levels of lysozyme when compared to the normals or to the patients with disseminated disease (Stage III). Using Student's "*t*" test the significance of the difference between these groups was $P < 0{\cdot}01$. In other words, elevated serum lysozyme in malignant melanoma patients is associated with localized disease. However, as can be seen from the diagram, the difference between the mean serum lysozyme in the Stage I cases and either the normals or the Stage III cases is partly due to a small number of Stage I cases with substantial elevation in serum lysozyme activity. Whether or not the subgroup has a better prognosis is the subject of prospective studies.

*Hypernephroma*

In these 25 patients, marked elevation of the serum lysozyme was found almost exclusively in those cases with disease confined to the kidney (see Fig. 2). However, in one patient with a single 1 cm lung metastasis (examined 3 months after nephrectomy) there was significant elevation. Two patients with localized primary tumours had abnormally low levels of lysozyme but both these cases had received preoperative irradiation to the renal area. The difference between

FIG. 2.—Serum lysozyme activity in patients with hypernephroma, showing significant elevation in those with disease confined to the kidney. Two cases, ○, had received irradiation to the renal area before the blood samples were taken. One patient, (●), with elevated lysozyme activity in his serum had only a single small static metastasis in one lung.

the two groups is significant at the 5% level even when the three aberrant values are included. The serum lysozyme levels in the normal controls are also included in this figure for comparison.

*Carcinoma of the breast*

Sera were obtained from patients at their first presentation at a breast clinic and were assayed for lysozyme activity. The patients were subsequently admitted to hospital for investigation, staging and treatment. Of the 97 sera tested, there were 84 untreated cases of breast carcinoma and 13 with benign breast lesions.

The 84 new cases of breast cancer were staged surgically, histologically and by intensive investigation including bone-marrow examinations, bone scanning, liver scanning and ultrasonography and a complete biochemical profile including serum calcium, alkaline phosphatase and urinary hydroxyproline excretion.

There were 20 cases with an isolated primary tumour (T+, No, Mo), 30 with histologically proven node metastases (T+, N+, Mo) and 34 who presented with evidence of distant metastases (T+, N+, M+).

The serum lysozyme results are shown in Fig. 3.

The mean serum lysozyme levels in cases with disease confined to the breast were significantly higher than those with either lymph node or distant metastases ($P < 0{\cdot}05$) but it can be seen from the diagram that the area of overlap was extensive and that this difference is due

FIG. 3.—Serum lysozyme activity in patients with benign (13 cases) or malignant (84 cases) breast lesions. The three benign cases with the highest lysozyme levels, ○, had inflammatory lesions, the remainder were typical cases of fibroadenosis. In the carcinoma patients all those with lysozyme levels above 12·5 $\mu$g/ml had no evidence of metastatic disease.

to a group of the T+, No, Mo cases with elevated lysozyme levels. However, as this diagram indicates, a serum lysozyme of above 12·5 $\mu$g/ml in a patient with a histologically proven primary breast carcinoma is associated with the absence of overt metastatic disease. The benign breast lesions seem to fall into two groups. Three cases with inflammatory diseases (2 degenerating cysts and 1 case of fat necrosis) had abnormally high lysozyme levels, whereas the remainder, with fibroadenosis, were within the normal range. In untreated patients with distant metastases the lysozyme levels were not significantly different from the normals and in those cases with axillary node involvement there was similarly no statistically significant elevation. Staging of the tumour alone revealed an association between tumour stage and serum lysozyme but only in those cases free from metastatic disease, i.e. the more extensive the primary tumour, the higher the serum lysozyme. This correlation was absent in the cases with detectable nodal or distant metastases. Examination of those cases with disease confined to the breast has failed to reveal any obvious association between histological appearance and the serum lysozyme.

*Release of lysozyme by cells from deposits of malignant melanoma*

Samples of tumour obtained at operation were washed, trimmed free of normal and necrotic areas and finely chopped with a pair of scalpel blades. A single cell suspension was then obtained by enzyme disaggregation using 0·1% trypsin and 0·1% collagenase. The enzyme treatment was continued at 37°C until all macroscopic fragments had disappeared (usually 1–1½ h). The resulting cell suspensions were washed twice in medium 199 containing 10% foetal bovine serum and once in serum-free medium. Finally the cells were resuspended in serum-free medium at $4 \times 10^6$/ml. Twenty-$\mu$l samples of these suspensions were then added to the wells of lysozyme assay plates (containing medium 199 as the buffer diluent) and were incubated for 24 h at 37°C in a 5% $CO_2$ atmosphere. Standard curves of normal human lysozyme were obtained on the same plates and the results were expressed as $\mu$g lysozyme/$10^6$ cells/24 h.

Cells from these suspensions were also cultured in RPMI1640 plus 10% foetal bovine serum in plastic culture flasks. After two days' incubation the cultures were washed and trypsinized for 5 min with 0·1% trypsin. The detached cells were then assayed for lysozyme release as above. Passaged tumour cells obtained in this way failed to release

FIG. 4.—Release of lysozyme from cell suspensions from biopsy specimens of malignant melanoma. The highest lysozyme levels were found in localized lesions and this was associated with the presence of macrophages. The results are expressed as $\mu$g lysozyme released/$10^6$ cells/day.

detectable lysozyme, whereas the cells not removed from the flasks by trypsin (morphologically these were macrophages) continued to release detectable lysozyme activity into the culture supernatant for several weeks.

Suitable suspensions of high viability were obtained from 16 cases of malignant melanoma. Detectable lysozyme release was present in 13 of these and in each the lysozyme was released from tumour macrophages and not from the malignant cells themselves. The data obtained from these cases are shown diagrammatically in Fig. 4. As this diagram shows, substantial release of lysozyme was only detectable in samples obtained from localized deposits, whereas the release of lysozyme by cells from distant metastases was minimal. This simple assay procedure may give useful semi-quantitative information concerning the degree of macrophage infiltration of a tumour. How well these *in vitro* results reflect the relative proportion of macrophages *in vivo* in a solid tumour mass is of course open to question. However, the value of lysozyme release for measuring macrophage contamination of a " tumour cell " suspension in this system was confirmed by the studies of Gauci and Alexander (1975) who examined the same cell suspensions for macrophage content using a specific anti-human macrophage serum and obtained similar results.

### DISCUSSION

If the levels of lysozyme in the serum of cancer patients are a reflection of host resistance, as seems to be the case in an animal model (Currie and Eccles, 1976) certain predictions can be made. Patients with localized primary tumours should have higher levels than both normal individuals and those with metastatic disease. The data obtained from patients with malignant melanoma, hypernephroma and breast carcinoma are in accord with such a prediction.

The breast carcinoma cases were staged surgically, histologically and by intensive investigation, and it can be seen that those women with a histologically proven primary breast carcinoma who presented with significant elevation in serum lysozyme (above 12·5 $\mu$g/ml) had no detectable metastatic disease. Any further conclusions about the prognostic significance of such an observation must obviously await a prospective controlled study. However, it is tantalizing to note that exactly half the patients with a localized primary breast carcinoma (NoMo) show a significant elevation in serum lysozyme.

Fogelson and Lobstein (1954) have examined the lysozyme content of whole blood in 77 normal individuals and 35 patients with localized and generalized carcinomatosis. They demonstrated that the cancer patients had statistically higher blood lysozyme activity than the normal individuals. However, they stated that the increase had no clinical or diagnostic significance. In patients with malignant tumours of the urinary tract, Kovanyi and Letnansky (1971) have shown significant elevation of lysozyme in both urine and serum. They claimed that the simultaneous estimation of urinary and serum lysozyme activity was a valuable diagnostic test for malignant disease in the genito-urinary tract. More recently Cooper *et al.* (1974) have examined serum lysozyme activity in patients with colo-rectal cancer before and after surgical treatment and indicated that patients with primary tumours had significantly higher serum lysozyme levels than normal controls and that surgical excision led to a significant fall in serum activity. There was also a suggestion that patients with metastatic disease had higher levels than those with localized tumours and long term follow-up studies showed that raised lysozyme levels may occur as a transient phenomenon in recurrent or metastatic disease. They speculated that the elevation of serum lysozyme activity in these patients may reflect a host reaction to the tumour. Subsequently Jedrzejczak and Siekierzynski (1975) criticized this series and stated that raised serum lysozyme levels in cancer patients may be due to increased granulocyte turnover associated with infection and are therefore of no diagnostic or prognostic value. In the animal studies of Currie and Eccles (1976) there was no evidence of any infectious process, and in the patients with malignant melanoma and breast carcinoma, those with the highest lysozyme levels were the patients with primary lesions, none of which were infected. Primary lesions in malignant melanoma are frequently infiltrated with macrophages (Currie, Lejeune and Fairley, 1971) and the production of increased amounts of lysozyme is, I suggest, most likely to be due to macrophages resident in the tumour mass or in the regional lymph nodes. Furthermore, single cell suspensions obtained from human malignant melanoma release lysozyme *in vitro* in a manner similar to that found in the rat tumours where it is associated with the presence of macrophages.

There is an apparent contradiction between the results obtained in the animal model systems (Currie and Eccles, 1976) and those seen in the cancer patients. In general, elevated lysozyme levels in the cancer patients were associated with early or localized disease whereas in the HSBPA rat sarcoma, for instance, the levels in the serum rose progressively with tumour growth. However, the HSBPA tumour is highly immunogenic, does not metastasize at any stage and seems to maintain its high content of macrophages throughout tumour growth. The lysozyme data from the cancer patients suggest that there may be a fall in the macrophage content of tumours as they grow and disseminate. Our studies suggest that localized tumours (malignant melanoma) have a higher macrophage content than do distant metastases. The rat sarcomata studied in our previous paper (Currie and Eccles, 1976) present distinctly different types of biological behaviour. The HSBPA sarcoma already mentioned rarely metastasizes and is associated with high serum lysozyme levels, whereas the highly metastatic tumour ($MC_3$) evokes only low serum levels. These rat sarcomata may be analogues of the various stages in the natural history of human " solid " tumours. A localized primary tumour is under some form of host restraint associated with large numbers of macrophages resident within the tumour mass and its draining regional lymph nodes. This macrophage response could account for the elevated serum lysozyme levels. The development of metastatic disease due to (or accompanied by) the failure of host resistance is associated with sparse macrophage infiltration of the tumour mass and therefore with lower levels of lysozyme in the serum.

The serum level of lysozyme is affected by many variables such as granulocyte kinetics, renal function and the total number of macrophages and monocytes in the body. Because of this it is unlikely to be of value as a screening test for detecting or even following the progress of cancer patients (Cooper *et al.*, 1974). However, in the presence of a histologically proven primary tumour it may be of value in staging, by identifying those patients in whom some form of host resistance associated with macrophage infiltration is still operating.

I am indebted to Dr Trevor Powles, The Royal Marsden Hospital, for access to the sera from patients with breast carcinoma and to Isobel MacCallum for expert technical assistance.

### REFERENCES

COOPER, E. H., TURNER, R., STEELE, L. & GOLIGHER, J.C. (1974) Blood Muramidase Activity in Colorectal Cancer. *Br. med. J.*, iii, 662.

CURRIE, G. A., LEJEUNE, F. & FAIRLEY, G. H. (1971) Immunization with Irradiated Tumour Cells and Specific Lymphocyte Cytotoxicity in Malignant Melanoma. *Br. med. J.*, ii, 305.

CURRIE, G. A. & ECCLES, S. A. (1976) Serum Lysozyme as a Marker of Host Resistance. I. Production by Macrophages Resident in Rat Sarcomata. *Br. J. Cancer*, **33**, 51.

FOGELSON, S. J. & LOBSTEIN, O. E. (1954) A Statistical Comparison of the Blood Lysozyme Activity of Normal Adults and of Patients with Localized and Generalized Carcinomatosis. *Amer. J. dig. Dis.*, **21**, 324.

GAUCI, C. L. & ALEXANDER, P. (1975) The Macrophage Content of Some Human Tumours. *Cancer Letters*, **1**, 29.

JEDRZEJCZAK, W. W. & SIEKIERZYNSKI, M. (1975) Serum Muramidase Activity in Untreated Cancer. *Br. med. J.*, i, 735.

KOVANYI, G. & LETNANSKY, K. (1971) Urine and Blood Serum Muramidase (Lysozyme) in Patients with Urogenital Tumours. *Eur. J. Cancer*, **7**, 25.

OSSERMAN, E. F. & LAWLOR, D. P. (1966) Serum and Urinary Lysozyme (Muramidase) in Monocytic and Monomyelocytic Leukaemia. *J. exp. Med.*, **124**, 921.